# Imbalance of placental regulatory T cell and Th17 cell population dynamics in the FIV-infected pregnant cat

**DOI:** 10.1186/1743-422X-9-88

**Published:** 2012-05-04

**Authors:** Crystal E Boudreaux, Lyndon B Chumbley, Veronica L Scott, Dwayne A Wise, Karen S Coats

**Affiliations:** 1 Department of Biological Sciences, Mississippi State University, P.O. Box GY,, Mississippi State, MS, 39762, USA

**Keywords:** Regulatory T cells, Th17 cells, Feline Immunodeficiency Virus, Placenta

## Abstract

**Background:**

An appropriate balance in placental regulatory T cells (Tregs), an immunosuppressive cell population, and Th17 cells, a pro-inflammatory cell population, is essential in allowing tolerance of the semi-allogeneic fetus. TGF-β and IL-6 are cytokines that promote differentiation of Tregs and Th17 cells from a common progenitor; aberrant expression of the cytokines may perturb the balance in the two cell populations. We previously reported a pro-inflammatory placental environment with decreased levels of FoxP3, a Treg marker, and increased levels of IL-6 in the placentas of FIV-infected cats at early pregnancy. Thus, we hypothesized that FIV infection in the pregnant cat causes altered placental Treg and Th17 cell populations, possibly resulting in placental inflammation.

**Methods:**

We examined the effect of FIV infection on Treg and Th17 populations in placentas at early pregnancy using quantitative confocal microscopy to measure FoxP3 or RORγ, a Th17 marker, and qPCR to quantify expression of the key cytokines TGF-β and IL-6.

**Results:**

FoxP3 and RORγ were positively correlated in FIV-infected placentas at early pregnancy, but not placentas from normal cats, indicating virus-induced alteration in the balance of these cell populations. In control cats the expression of IL-6 and RORγ was positively correlated as predicted, but this relationship was disrupted in infected animals. TGF-β was reduced in infected queens, an occurrence that could dysregulate both Treg and Th17 cell populations. Co-expression analyses revealed a highly significant positive correlation between IL-6 and TGF-β expression in control animals that did not occur in infected animals.

**Conclusion:**

Collectively, these data point toward potential disruption in the balance of Treg and Th17 cell populations that may contribute to FIV-induced inflammation in the feline placenta.

## Background

Maternal tolerance of the semi-allogeneic fetus is accomplished, in part, by the expression of cytokines and other immunoregulatory molecules at the maternal-fetal interface which modulate appropriate placental immunology [1,2]. The Th1/Th2 paradigm, which assumed that Th2 (anti-inflammatory) cytokines dominate the maternal-fetal interface during most of pregnancy while Th1 (pro-inflammatory) cytokines are suppressed until the approach of parturition [3], has been recently succeeded by a Th1/Th2/Th17 and regulatory T cell paradigm [4] that allows for a cytokine milieu during various stages of pregnancy which does not fit the traditional Th1/Th2 model. Clearly, placental immunology is highly complex and temporal, coincident with changes in cell populations and immunomodulation as pregnancy progresses from implantation to onset of labor (reviewed in [5]).

Tregs and Th17 cells differentiate from a common T helper progenitor cell. TGF-β causes the proliferation of both Treg and Th17 cells [6,7]. During inflammation, enhanced production of IL-6 inhibits the induction of FoxP3, halting the generation of Tregs, and activates the expression of RORγ, driving the proliferation of Th17 cells [8]. Th17 cells further enhance the inflammatory response by releasing the cytokines IL17, IL-6, TNFα, and IL-22. Thus, IL-6 levels dictate a pro- or anti-inflammatory cellular response and an inverse relationship between Tregs and Th17 cells [8]. Disruption in cytokine expression potentially perturbs the balance in the two cell populations.

Several investigators have correlated increased numbers of activated Treg cells in the periphery and decidua with successful pregnancies, while a reduced number of Tregs accompany failed pregnancies [9-11]. The inflammatory cytokine IL-17, a product of Th17 cells, was found to localize in cytotrophoblast and syncytiotrophoblast cells in the deciduas of unexplained recurrent spontaneous abortions (URSA) and normal early pregnant women, but IL-17 was significantly higher in the URSA group [12]. This finding suggests that Th17 cells may play a role in pregnancy failure.

The complexity of placental immunology confounds the ability to define the causal relationship between aberrant placental immunology and perturbed pregnancy. Importantly, HIV infection in pregnant women has been associated with increased reproductive failure [13,14], and there is evidence that the virus alters placental cytokine expression, promoting mother-to-child transmission [15]. The need for additional study is apparent; yet, the inability to obtain human tissues at will to conduct these investigations highlights the value of an animal model.

We use the FIV-infected cat model to evaluate parameters of lentivirus-induced placental inflammation. Our previous data suggest a pro-inflammatory placental microenvironment at early pregnancy in the infected cat, based on the ratio of pro- to anti-inflammatory cytokines, expression of IL-6 [16], and the likely decreased population of Tregs [17]. In the present study we hypothesized that FIV infection in the pregnant cat causes altered placental Treg and Th17 cell population dynamics, allowing placental inflammation that may compromise pregnancy. Placental immunology is distinctly different at early and late stages of pregnancy; therefore, it was important to evaluate the impact of FIV infection in the feline placenta at these two time points. Our first objective was to localize the two cell populations by labeling parallel sections with either FoxP3 or RORγ-specific antibody and comparing both to a parallel specimen immunolabeled with anti-relaxin antibody. As relaxin is produced by trophoblasts, its presence demarcates the maternal-fetal interface. The two cell populations were localized to this region. Our second objective was to quantify the expression of Treg marker FoxP3 and Th17 marker RORγ in placental samples from FIV-infected and control queens at early gestation by immunofluorescence confocal microscopy. FoxP3 expression was significantly reduced in infected tissues while RORγ expression was unaffected. FoxP3 and RORγ were positively correlated in FIV-infected placentas, while in control placentas no correlation occurred. The third objective was to evaluate expression of the key cytokines that drive Treg and Th17 proliferation, TGF-β and IL-6. TGF-β was significantly reduced, and the normal positive correlation in co-expression between the two cytokines was disrupted in infected tissues. Finally, we correlated expression of IL-6 with RORγ. As an enhanced level of IL-6 is indicative of inflammation, its expression should be inversely correlated to Treg function and positively correlated with Th17 function. In control cats the expression of IL-6 and RORγ was positively correlated as predicted, but this relationship was disrupted in infected animals. Collectively, the data suggest that FIV-induced immunopathology may include perturbation of Treg/Th17 cell balance at early pregnancy. This report supports our prior evidence of a virus-induced, pro-inflammatory placental microenvironment at early pregnancy.

## Methods

### Animals and virus

Cats (*Felis domesticus*) were specific pathogen free (SPF), reproductively-mature female animals purchased at age less than 12 months. The animals were obtained from a commercial cattery (Liberty Research, Inc.). Ten cats were inoculated intravenously with 1 cc a feline plasma pool containing FIV-B-2542 at approximately 1×10^4^ copies per ml; ten cats were uninoculated controls. Whole blood (15 ml) was collected into Vacutainer^®^ tubes at biweekly to monthly intervals until delivery of kittens. Serum, plasma, and peripheral blood leukocytes (PBLs) were collected. Confirmation of infection was performed by standard PCR and serology [18]. Queens were allowed to breed naturally with SPF males. Fetuses were delivered by cesarean section immediately after pregnancies were confirmed by ultrasonography at week 3–4 gestation (early term) or at 8 weeks gestation (late term). The time of FIV inoculation to delivery ranged from approximately 9.5 to 13.5 months (mean 11.14 months) for the early-gestation study and 4.7 to 14.1 months (mean 9.5 months) for the late-gestation study. Fetal and placental tissues were collected from all animals, snap frozen in liquid nitrogen, and frozen at −80°C. Infected animals (n = 10) used were euthanized following delivery. Control (n = 10) cats were spayed and released for adoption after recovery. Animal protocols were approved by the Institutional Animal Care and Use Committee of Mississippi State University. Placental samples used for confocal analysis and immunohistochemistry and qRT-PCR analyses are shown in Tables 1 and 2 respectively.

**Table 1:** Placental samples included in confocal analyses

**Placenta Number Early Pregnancy**	**Queen FIV Status**	**Placental FIV Status**	**Fetal Outcome**	**Placenta Number Late Pregnancy**	**Queen FIV Status**	**Placental FIV Status**	**Fetal Outcome**
7824A	-	-	V	13671 AP	-	-	V
7824B	-	-	V	13671 DP	-	-	V
7824 C	-	-	V	13668 AP	-	-	V
2779A	-	-	V	13668 BP	-	-	V
2779E	-	-	V	9746 AP	-	-	V
6108A	-	-	V	9746 DP	-	-	V
8291A	-	-	V	9801 AP	-	-	V
8291B	-	-	V	9801 BP	-	-	V
9784B	-	-	V	9674 AP	+	+	V
6062B	+	+	V	9809 A	+	+	V
6062 C	+	+	V	13226 AP	+	+	NV
8035B	+	+	NV	13226 BP	+	+	NV
1893B	+	+	NV	9730 R1	+	+	NV
1893 C	+	+	V	9813 R1	+	+	NV
1126A	+	+	V	9813 R2	+	+	NV
1126D	+	+	V				
5111A	+	+	V				
5111 C	+	+	NV				
0866B	+	+	V				

V = viable fetus.

NV = non-viable fetus.

R = resorption.

**Table 2:** Placental samples included in Treg expression analyses

**Fetus Number**	**Queen FIV Status**	**Placental FIV Status**	**Fetal Outcome**
8059A	-	-	V
8059B	-	-	V
8059 C	-	-	V
8059D	-	-	V
6108A	-	-	V
6108B	-	-	V
6108 C	-	-	V
7824A	-	-	V
7824B	-	-	V
7824 C	-	-	V
6062A	+	+	V
6062B	+	+	V
8035A	+	+	V
8035 C	+	+	NV
1126A	+	+	V
1126B	+	+	V
5111B	+	+	V
5111 C	+	+	NV
0866A	+	+	V
0866B	+	+	NV

V = viable fetus.

NV = non-viable fetus.

All surgical procedures were performed under appropriate anesthetic protocols. All queens were premedicated with acepromoazine (0.05 mg/kg) and burophanol (0.2 mg/kg) administered intramuscularly twenty minutes before induction. A 50:50 mixture of diazepam (5 mg/ml) and ketamine (100 mg/ml) was used for induction at an intravenous dosage of 0.1-0.14 ml/kg. The cat was then intubated and maintained under anesthesia with 1-2% isoflurane. Intravenous fluids were provided at a rate of 10 ml/kg/h. After collection of reproductive tissues and blood samples, all queens were euthanized (Beuthanasia solution 1 ml/lb IV) while under anesthesia. This method was consistent with the recommendations of the Panel on Euthanasia of the American Veterinary Medical Association. Animal protocols were approved by the Institutional Animal Care and Use Committee of Mississippi State University.

### Detection of virus in tissue

Placental tissues were evaluated for FIV provirus using standard PCR or for viral RNA targeting FIV gag using qPCR according to the published protocols [17-19].

### Purification of RNA from feline placental tissues and conversion to cDNA

Random sections of tissue were obtained from whole placenta. TRIzol Reagent (Invitrogen, Carlsbad, CA) was used to purify RNA from these tissues. RNA was purified from the aqueous phase as described [20]. Concentrations were determined using a NanoDrop 1000 (Thermo Scientific, Waltham, MA). Purified RNA was reverse transcribed using High Capacity cDNA kit (Applied Biosystems). Reverse transcription reactions (20ul) were performed using the following protocol: 25°C for 10 min, 37°C for 120 min, 85°C for 5 sec.

### Quantification of cytokines and Treg markers by real time PCR

Complementary DNA obtained from the High Capacity cDNA reactions were used in gene expression analysis. Primetime assays (Integrated DNA Technologies, Coralville, Iowa) were used to evaluate the expression levels of IL-6 and TGF-β, along with the housekeeping gene β-actin. The primer/probe combination for use in real time analysis of TGF-β (GenBank accession number AY425617) was as follows: probe (/56-FAM/AGC AAT AAT/ZEN/TCC TGG CGC TAC CTC AGC A/3IABkFQ/), forward primer (AGC ACG TGG AGC TGT ACC AGA AAT), and reverse primer (TCC AGT GAC ATC AAA GGA CAG CCA). Primer/probe combinations used for IL-6 and β-actin were previously described [21]. Probes for target genes were labeled at the 5’ and 3’ ends with 6-carboxyfluorescein (FAM) and Iowa Black ^®^ FQ, respectively. Simplex reactions were performed with a target gene and the housekeeping gene using an ABI thermocycler (Applied Biosystems, Carlsbad, CA) with the following protocol: Step 1- 50°C, 2 min and 95°C, 10 min; Step 2–40 cycles (95°C for 15 sec and 60°C for 1 min.) [16] . Each reaction well contained 10ul of the 2X Taqman gene expression master mix, 1ul of 20X TaqMan gene expression assay and approximately 6ug of cDNA. For every placental RNA sample, parallel reactions were run in duplicate on separate plates for each gene. Serially-diluted, pooled RNA from control cats was used to generate a standard curve for simplex reactions. Standard curves were used to calculate the correction coefficient for each target. Differences in the amount of template cDNA in each reaction were corrected by the cycle threshold (Ct) value for β-actin. Comparisons of Treg markers between infected versus uninfected placentas were analyzed statistically using ANOVA. Correlation analyses were done using simple regression analyses (http://asuc.org/index.php/student-resources/mentorship-program). Differences were considered significant at p ≤ 0.05.

### Localization of Tregs and Th17 cells in placental tissues using immunohistochemistry (IHC)

An IHC protocol developed by our laboratory for detection of specific cell populations in frozen feline placental sections [22] was used to localize Treg and Th17 cells. Rabbit polyclonal antibodies (Abcam, Cambridge, MA,1:500) to FoxP3 and RORγ were used to identify Treg and Th17 cells, respectively. The maternal-fetal interface was determined using a rabbit polyclonal antibody to porcine relaxin (1:500) (a gift from Dr. Peter Ryan, Mississippi State University Department of Animal and Dairy Sciences) [23,24]. Parallel sections were stained sequentially for relaxin, FoxP3, RORγ, and a universal negative control. Tissues were incubated with ready to use secondary antibody, goat anti-rabbit IgG, poly-horseradish peroxidase (HRP) (Chemicon International Inc., Temecula, CA.) and developed with 3,3’-diaminobenzidine (DAB, Invitrogen). Tissues were counterstained with Mayer hematoxylin and dehydrated.

### Immunofluorescence staining

Snap frozen placental tissues were embedded in OCT (Tissue-Tek^®^, Sakura Finetek, Torrance, CA.) and sectioned to a thickness of 4um using a cryostat. Five random sections for each placenta collected from the maternal/fetal interface were placed on poly-L-lysine coated slides and allowed to dry. Tissues were fixed in acetone (100%) for 15 minutes at room temperature and then treated with 0.01% Triton-X-100 to permeabilize the tissue. Sections were blocked with feline IgG (0.1 mg/ul) and 5% non-fat milk, and incubated at 4°C for at least 1 hour. Sections were incubated with either rabbit polyclonal antibody to RORγ (Abcam, Cambridge, MA; dilution 1:500) or rabbit polyclonal antibody to FoxP3 (Abcam, Cambridge, MA; dilution 1:500) for 2 h at 4°C. Secondary antibody, goat anti-rabbit IgG (H + L) fluorescein conjugated (CHEMICON, Temecula, CA, 1:500), was applied and incubated 45 minutes to 1 h at 4°C. Parallel sections were treated with isotype-matched control antibodies (Dako North America Inc., Carpinteria CA.) conjugated with appropriate fluorochromes to assure that reactivity was not a result of non-specific binding. Sections were mounted with Vecta shield containing DAPI (Vector Laboratories, Inc.).

### Confocal microscopy

The fluorescence intensity was acquired using a Zeiss LSM 510 confocal laser scanning microscope (Carl Zeiss Microimaging, Inc, Thornwood, NY) with an inverted Zeiss Axiovert 200 M light microscope and a plan apochromat 40 X/1.30 NA objective lens. A DAPI/fluorescein filter set was used in single channel mode imaging. Excitation wavelengths of 405 nm and 488 nm were used and Band Pass (BP) Emission wavelengths of 420–480 nm (Blue) and Long Pass (LP) 505 nm (Green) were acquired at 1024 × 1024 pixel format for imaging purposes. Fluorescence intensity was collected from 10 randomly selected fields of view per section. A ratio of FoxP3 or RORγ intensity versus DAPI intensity was calculated to quantify the number of stained cells/total cells. Background fluorescence was acquired for each sample using the secondary antibody.

### Statistical analyses

Statistical analyses of mean fluorescence intensity for targeted cell populations (FoxP3 and RORγ) between FIV control and infected cats were done using ANOVA or Wilcoxon two-independent sample *T* test. Simple regression analysis (http://www.socr.ucla.edu/htmls/SOCR_Analyses.html) was used to determine correlations. Differences were considered significant at p ≤ 0.05.

## Results

### FIV infection status of placental tissues

All inoculated animals became infected with FIV [18,19], and FIV provirus or viral RNA was detected in all placental tissues tested [17]. Fetal outcome in early [19] and late-term [18] pregnancies was reported previously; non-viability was significantly higher in FIV-infected queens at both stages of pregnancy. Placental samples and infection status are listed in Tables 1 and 2. Table 1 identifies placental samples from early and late term gestation included in the confocal analyses used to quantify RORγ and FoxP3 in the infected and control groups. Table 2 identifies placental samples from early term gestation included in the Treg expression analyses.

### Localization of Tregs and Th17 cells at the maternal-fetal interface

**Figure 1:**
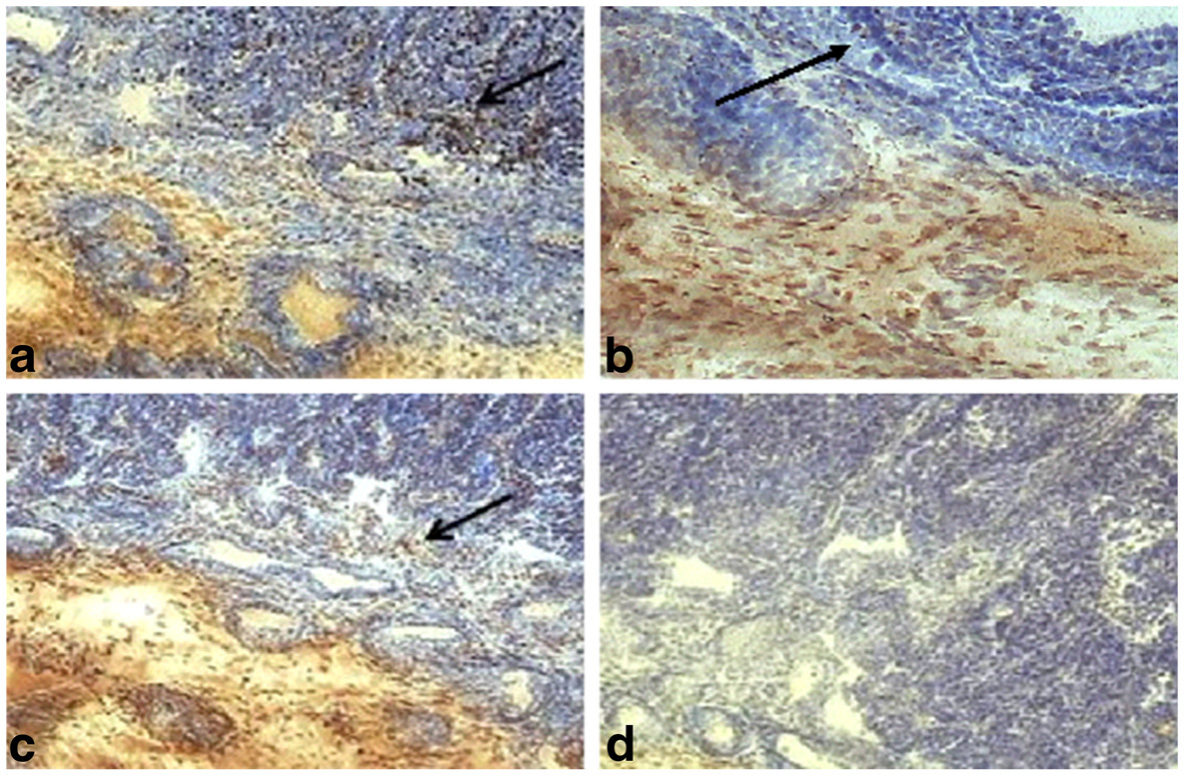
**Immunohistochemical localization of relaxin, FoxP3, and RORγ at the maternal-fetal interface of a representative early term placental sample.** Relaxin localized in the placental trophoblast at the maternal-fetal interface (arrow) (**a**). FoxP3 and RORγ co-localized at the maternal-fetal interface (arrows) (**b,c**). A universal negative control was included to assure no nonspecific binding occurred (**d**). (20X magnification).

Tregs and Th17 cells were localized by comparing parallel tissue sections immunolabeled for relaxin (Figure 1a), FoxP3 (Figure 1b), and RORγ (Figure 1c). A specimen treated with the universal negative control reagent (Figure 1d) did not label, showing that non-specific binding of the secondary antibody did not occur.

### Quantification of RORγ and FoxP3 staining in FIV-infected and control cats

**Figure 2:**
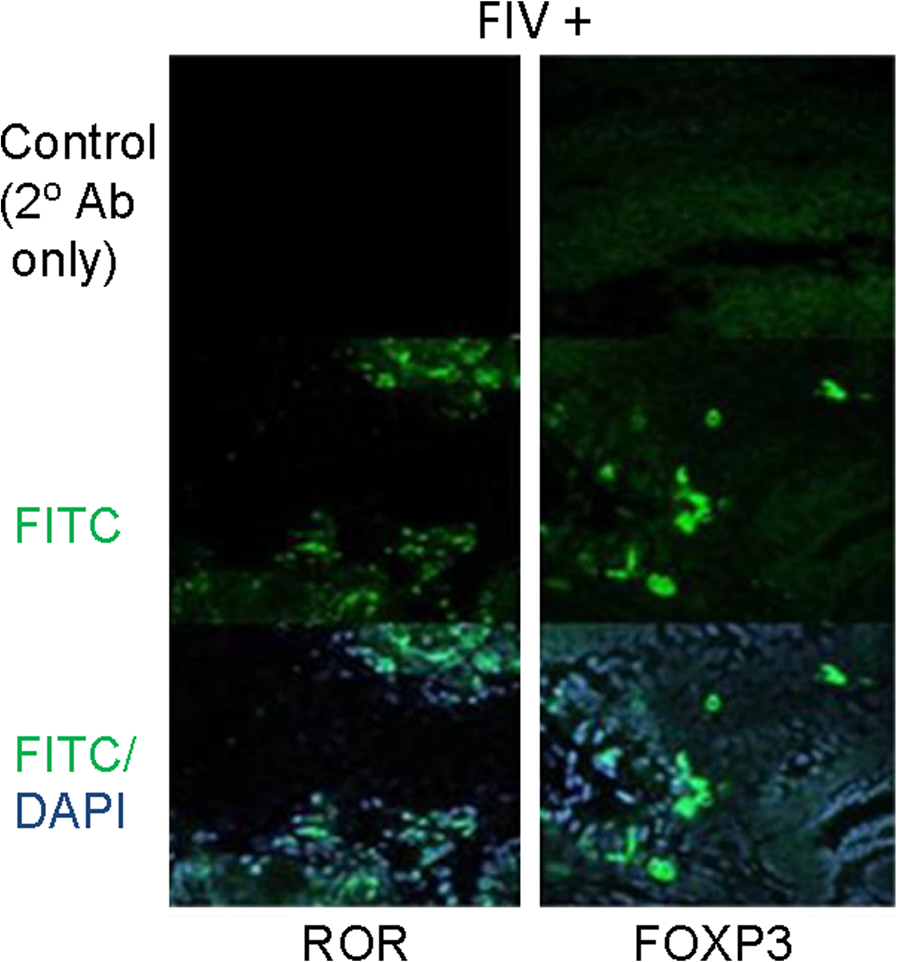
**Immunofluorescence labeling of RORγ and FoxP3 at the maternal fetal interface of tissue from representative infected cat.** Negative controls included no primary antibody (top panels). RORγ and FoxP3 (green) were detected using polyclonal rabbit anti-FoxP3 or anti-RORγ antiserum followed by goat anti-rabbit IgG (H + L) fluorescein conjugate (middle panels). Cells were counterstained with DAPI (blue) (bottom panels). Cells were viewed by confocal laser scanning microscopy using a 40x oil immersion objective.

Immunofluorescence-labeling of FoxP3 and RORγ expressing cells, Treg and Th17 cells, respectively, at the maternal-fetal interface is shown in representative FIV infected samples (Figure 2). Negative control reactions (Figure 2, top panel) showed minimal background fluorescence, validating the specificity of the primary antibodies. Reactivity to FoxP3 or RORγ is shown (middle panel). The intranuclear expression of FoxP3 and RORγ was evident when labeled cells were stained with DAPI (bottom panel).

**Figure 3:**
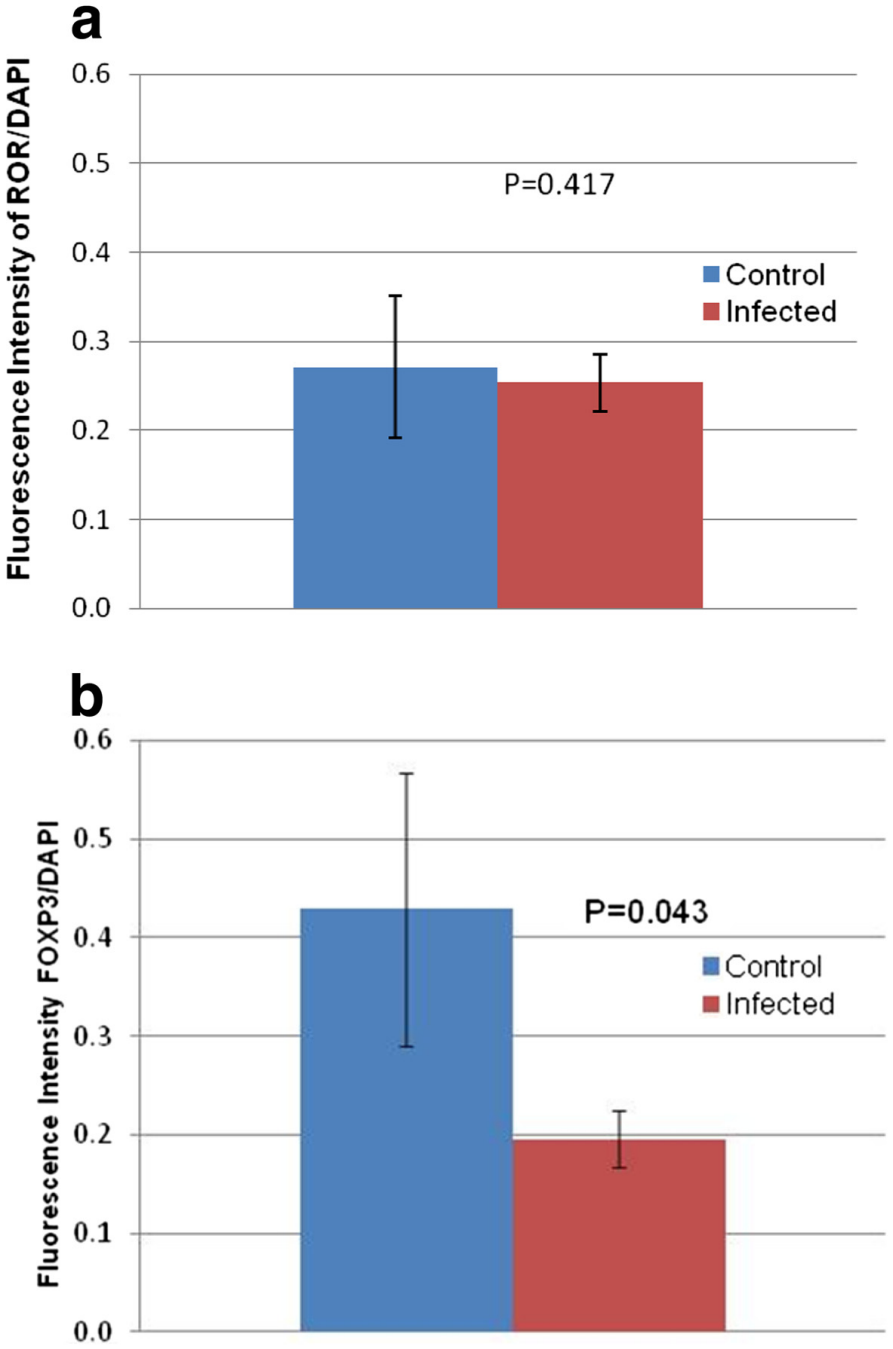
**Quantification of RORγ and FoxP3 staining in FIV infected and control cats.** Mean fluorescence intensity of FoxP3 and RORγ, which label Treg and Th17 cells, respectively, were measured in both control and infected cats. Values are bracketed by standard error of the mean. Control (n = 9) versus infected (n = 11) samples were evaluated. P values < 0.05 were considered significant.

**Figure 4:**
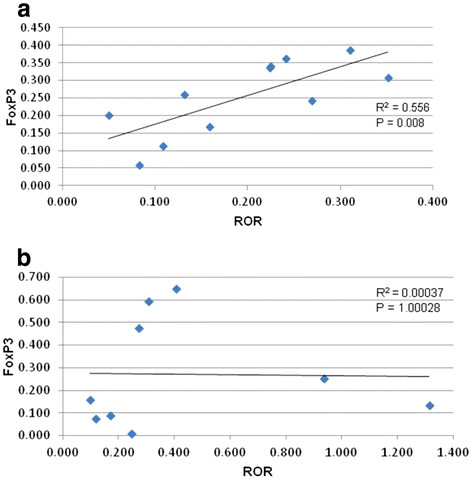
**Correlation of expression of FoxP3 and RORγ.** Correlation of FoxP3 to RORγ in infected (**a**) and control (**b**) cats at early gestation. Analysis was performed using a simple regression analysis. P values <0.05 were considered significant.

The cell populations were quantified by measuring the mean fluorescence intensity of the respective cell markers relative to the total cell population. Based on the detection of RORγ, there was no significant difference in the Th17 population between infected and control groups at early gestation (p = 0.417) (Figure 3a). However, the FoxP3 measurement was significantly lower in the infected than the control group (p = 0.043), suggesting a decreased population of Treg cells in placentas from infected animals. FoxP3 and RORγ expression positively correlated in FIV-positive cats at early gestation (p = 0.008). However, the two markers were independent in control animals (p = 1.00), (Figure 4 a and b). We detected no significant differences between specimens collected from viable and non-viable placentas. Therefore, those comparisons are not presented separately.

### Expression of IL-6 and TGF-β in placental tissues

**Figure 5:**
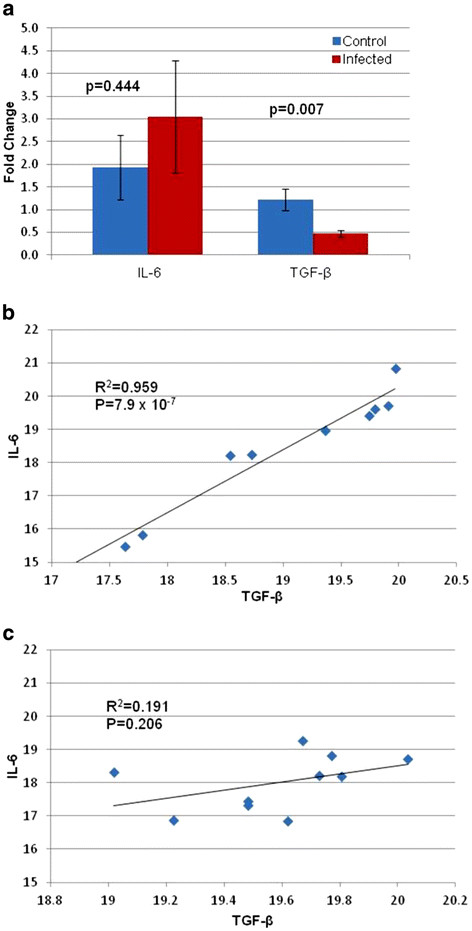
**Expression of IL-6 and TGF-β in placental tissues.** Fold change for IL-6 and TGF-β were calculated in control versus infected animals (**a**). The expression of IL-6 and TGF-β were correlated in control (**b**) (n = 10) and infected (**c**) (n = 10) placental tissues at early gestation. The data were analyzed using an ANOVA (A) or simple regression analysis (B,C). P values <0.05 were considered significant.

Differences in the expression of IL-6 and TGF-β were determined using the comparative Ct method with mean values for each cytokine from the controls used as the references. The fold change for IL-6 from infected animals was 3.05, while that from control animals was 1.93. However, perhaps due to large variation in this small sample size, the data did not reach the level of significance (p = 0.444). TGF-β expression was significantly reduced (p = 0.007) in infected tissues (fold change = 0.477) as compared to control samples (fold change = 1.23) (Figure 5a). Co-expression of the two cytokines was subjected to correlation analysis. Control animals exhibited a highly significant positive correlation in the expression of the two cytokines (P = 7.0 × 10^-7^) (Figure 5b), while infected animals were neither positively nor negatively correlated (Figure 5c). We detected no significant differences between specimens collected from viable and non-viable placentas. Therefore, those comparisons are not presented separately.

## Conclusion

Tregs accumulate in the human decidua during early, normal pregnancy, while Th17 cells are depressed at this stage. The ratio of these cells is reversed as parturition approaches, allowing the inflammatory environment that promotes the onset of labor and expulsion of the fetus [3]. Likewise, Th17 cells often dominate in cases of failed pregnancy or placental infection [12,25], illustrating the importance of the balance in these cell populations to pregnancy outcome. The data concerning a role for Tregs and Th17 cells in the feline placenta is extremely limited. Thus, it was important to confirm the presence of both populations and the location of these cells within the tissue. We did so by comparing parallel sections immunolabeled for expression of FoxP3 and RORγ protein to sections labeled for relaxin. Relaxin is a protein hormone produced by decidual cells that causes cervical ripening, pelvic elasticity, and induction of labor late in human pregnancy, in part by inducing the expression of key inflammatory cytokines from decidual cells [26]. In the cat, relaxin is known to be expressed by trophoblasts [27]. Thus, expression of relaxin can be used to demarcate the maternal-fetal interface. We found that Tregs and Th17 cells co-localize at the maternal-fetal interface in the cat.

In this study, we used placental tissues from FIV-infected and control queens to begin to evaluate how these cell populations may be involved in lentivirus-induced placental inflammation in the cat model. In previous studies we reported a pro-inflammatory placental environment and high levels of reproductive failure in the FIV-infected cat model of mother-to-child transmission of lentiviruses [17]. We found evidence of virus-associated depression of the Treg population at early pregnancy by qPCR analysis of expression of FoxP3 mRNA [17]. In the present study, FoxP3 protein was likewise depressed in the infected group, providing additional evidence of Treg depletion. On the other hand, RORγ was constant between infected and controls animals, suggesting no viral effect on this T cell population.

Treg and Th17 cell populations are normally negatively correlated with reciprocal pathways for immunosuppression or inflammation [28,29]. TGF-β and IL-6 facilitate the proliferation of Tregs and Th17 cells by activating their intranuclear transcription factors FoxP3 and RORγ, respectively. Although we predicted a negative correlation between FoxP3 and RORγ in control tissues given their usual reciprocal relationship in the periphery, expression of the two markers was independent in the control, correlating neither positively nor negatively. However, we detected a significant positive correlation of FoxP3 and RORγ co-expression at early pregnancy in the infected group. Given the role of these T cell populations during maintenance of pregnancy, we suspect that an imbalance of Treg and Th17 cells contributes to the fetal demise in FIV-infected cats at early pregnancy that we reported previously [19].

Proliferation of Tregs depends on the continuous production of TGF-β, an anti-inflammatory cytokine, by the Treg population. TGF-β normally drives the differentiation of both Tregs and Th17 cells from progenitor CD4+ T cells. Generation of Th17 cells requires the additional presence of IL-6 and IL-21 [30]. In this study, we found that FIV infection alters cytokine expression in the placenta. A significant decrease in TGF-β in placentas from the infected group was detected, possibly explaining the decrease in the placental Treg population in infected cats at early pregnancy that we reported here and previously [17]. However, Th17 cells are likewise dependent upon TGF-β to initiate their differentiation from progenitor T cells. We found significantly increased levels of IL-6 expression in early-term placentas in an earlier study [16]. Measuring fold change in IL-6 expression between control and infected tissues in the present study, the increase in IL-6 that was noted in infected tissues did not reach the level of significance due to large variation in this small sample size. This finding is consistent with the unchanged RORγ expression that we detected between the two groups. The detection of a positive correlation between the two cytokines in control animals while no significant correlation occurred in infected animals supports a virus-induced disruption in the normal balance between these two cytokines. Collectively, the data provide evidence that altered expression of key cytokines accompanies virus infection, likely contributing to the Treg/Th17 imbalance that occurs in infected animals.

This report provides additional evidence that FIV infection reduces the Treg population at the maternal-fetal interface in the cat model while leaving the Th17 population unchanged. This imbalance in the normal ratio of these cell populations would likely predispose a pro-inflammatory placental environment that would promote the excessive reproductive failure that we previously reported in this group of animals [19]. Currently, immunomodulator profiles from laser capture microdissected Treg and Th17 cells and adjacent placental microenvironments are being evaluated in the FIV-infected cat model. These profiles may illuminate the functional effects of viral infection on these immunologically-important placental cell populations.

## Competing interests

The authors declare they have no competing interests.

## Authors’ contributions

CEB conducted all experiments, analyzed data, and wrote the manuscript. LBC assisted with tissue sectioning. VLS provided instruction and some analysis with qPCR. DAW provided advice regarding confocal microscopy and data interpretation. KSC secured funding, supervised the project, assisted with data interpretation, and edited the manuscript. All authors read and approved the final manuscript.
